# Dysbiotic microbiota in autistic children and their mothers: persistence of fungal and bacterial wall-deficient L-form variants in blood

**DOI:** 10.1038/s41598-019-49768-9

**Published:** 2019-09-16

**Authors:** N. Markova

**Affiliations:** Institute of Microbiology, Department of Infectious Microbiology, Bulgarian Academy of Sciences, Acad. G. Bonchev str. 26, Sofia, 1113 Bulgaria

**Keywords:** Predictive markers, Prognostic markers, Diagnostic markers, Infection

## Abstract

Based on our hypothesis for existing microbiota of wall-deficient variants (L-forms) in human blood, we created an innovative methodology, which allowed for the development of L-form populations from blood of all investigated people. In contrast to healthy controls, blood L-forms from autistic children and their mothers converted under appropriate conditions of cultivation into detectable opportunistic bacteria and fungi, а process demonstrated by light and transmission electron microscopy. It can be distinguished into two types of states – “eubiotic” blood microbiota in healthy individuals, and “dysbiotic” in autistic children and their mothers. Remarkably, the unifying finding for autistic children and their mothers was the presence in blood of wall-free variants from life-cycle of filamentous fungi. Increased specific IgG, IgM and IgA, together with typical mold growth were a decisive argument for proven presence of *Aspergillus fumigatus* in almost all of the autistic children. As it was demonstrated in our previous study, filterable L-forms can be transmitted by vertical pathway from mother to child before birth. Thus, it can be suggested that autistic children may be born already colonized with fungi, while a “silent aspergillosis” could contribute or even be a leading cause for neurodevelopmental disorders in the early childhood.

## Introduction

Host microbiota can have a great impact on immune system and health^1,2^. Recently we reported about the presence of cell wall deficient bacterial variants (L-forms) in human blood. On the basis of known facts about their unique biology and persistence ability, as observed in our studies and those of other authors, we created a concept of blood L-form microbiota, which leads to novel insights with relevance to the human health or disease^3–6^. Domingue suggests that L-forms play a role in persistence and human pathology^7,8^. Beliefs that L-form microbiota may influence the host immune system and contribute to systemic inflammations, open new alternatives for understanding human pathophysiology as well as the pathogenesis of diseases with unknown origin.

Autism belongs to the group of neurodevelopmental disorders which are diagnosed mainly during early childhood. Centers for Disease Control and Prevention in the United States report a dramatic increase in autism’s prevalence by 15 percent (1 in 59 children, respectively 1 in 37 boys and 1 in 151 girls) for 2018^9^. Despite intense research work concerning pathogenesis of autism, the factors causing or influencing this state remain unclear. It is assumed that appearance of autism can depend on exposure to some endogenous or exogenous influence, be it infection or some chemical or physical agent^10^. Among the studied and discussed factors are immunological abnormalities, systemic infections and inflammations during the perinatal period or the period of early childhood^11–15^. Whether neurodevelopmental disorder in autistic children is primary per se or is a consequence of unrecognized persistent infections of fungal or bacterial origin, is not clear. Systemic microbial persistence can be suspected to be a contributing factor to autism but the causing microbes are difficultly proven with conventional approaches. That is why their presence and significance have often been questioned. Cell wall deficient variants (L-forms), both of bacterial or fungal origin may be suspected as possible persisters which can play role in these infections. In this respect, “L-form theory” proposes that an organism having both a pathogenic (walled) and a nonpathogenic (cell wall free) variant could provide a mechanism to explain the processes which govern the hidden/unrecognized microbial persistence. On the basis of accumulated knowledge about the unique biology/nature of cell wall deficient microbes, we have developed an innovative methodology for evaluation of human blood microbiota, which would be of importance for diagnosis of hidden latent infections^3–5^.

The goal of the current study was to isolate cell wall deficient variants (L-forms) from blood of autistic children, their mothers and control healthy persons, to observe and analyze their morphological transformations and characteristics, as well as to identify them after recovering of their cell walls.

## Results

### Development of blood L-form population in broth

**Figure 1:**
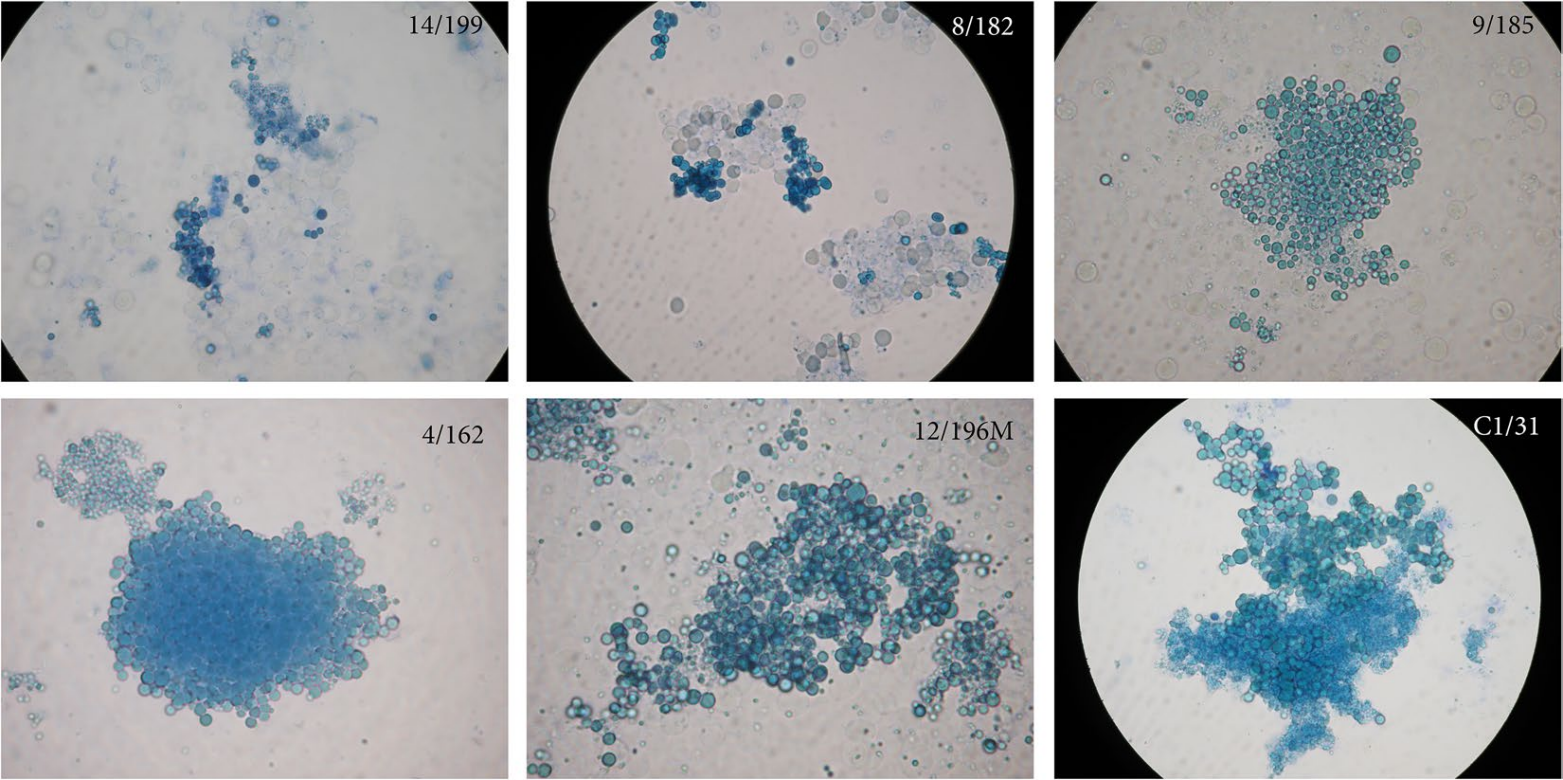
Representative development of L-form population (cell wall-deficient variants) in broth (TSB) inoculated with blood from autistic children (14/199, 8/182, 9/185, 4/162), a mother (12/196 M) and a control healthy person (C1/31). Native preparations contrasted with methylene blue - spherical and granular L-bodies. Magnification: 1000x.

Development of L-form population (microbial wall-deficient variants) in broth inoculated with blood was observed in all investigated people - children with ASD (Table 1), their mothers (Table 2) and 6 control healthy persons. Due to the innovative methodology concerning special techniques and intervals of sub-cultivations, the “non- cultivable elements” from blood were successfully adapted in broths, where they developed as replicable L-forms (cell wall- deficient variants). The sub-cultivation intervals were conducted in accordance to the periodic observations of native preparations and recognition of the phases of microbial transformation. As seen in Fig. 1, the observed populations of spherical and granular bodies were with typical characteristics for L-forms.

**Table 1:** Isolation of bacterial and fungal cultures from blood and urine of children with autism spectrum disorders (ASD).

ASD Patient	Age	Gender	Blood isolates				Urine isolates
			Bacterial cultures	Yeast cultures	Filamentous fungi -fungal elements in broths (+) –isolated culture (*)	*Aspergillus fumigatus*- specific IgG;IgM; IgA (Ref. titer <1:80)	Bacterial and yeast cultures
1/156	99 y.o.	m	*Enterococcus agglomerans*	*Candida parapsilosis Cryptococcusalbidus*	(+) (*)	(1:160)	ND
2/158	44 y.o.	m	*Rhizobium radiobacter*	*Candida parapsilosis*	(+) (*)	(1:640)	ND
3/160	111 y.o.	f	*Enterococcus faecalis*	*Candida parapsilosis*	(+) (*)	(1:320)	*Enterococcus faecalis Pseudomonas aeruginosa Rhodotorula mucilaginosa*
4/162	112 y.o.	m	—	*Candida parapsilosis*	(+) (*)	(1:640)	−
5/164	99 y.o.	m	—	*Candida parapsilosis*	(+) (*)	(1:320)	ND
6/178	55 y.o.	m	*Enterococcus faecalis*	*Cryptococcus albidus*	(+) (*)	ND	ND
7/180	44 y.o.	m	*Pseudomonas aeruginosa Morganella morganii*	*Candida parapsilosis*	(+) (*)	(1:320)	*Pseudomonas aeruginosa Morganella morganii Enterococcus faecalis Candida parapsilosis*
8/182	44 y.o.	f	−	−	(+) (*)	(1:160)	*Pseudomonas aeruginos Enterococcus faecalis*
9/185	44 y.o.	m	−	−	(+) (*)	(1:320)	−
10/188	33 y.o.	m	−	−	(+) (*)	(1:640)	ND
11/190	44 y.o.	m	−	*Rhodotorula mucilaginosa*	(+) (*)	(1:160)	−
12/194	66 y.o.	m	*Chryseobacterium indologenes*	−	(+) (*)	(1:320)	*Proteus mirabilis*
13/197	66 y.o.	m	−	−	(+)	(1:80)	*Acinetobacter spp*.
14/199	33 y.o	m	*Brevibacterium casei*	−	(+) (*)	(1:160)	ND
15/201	112 y.o.	m	*Aeromonas sobria*	−	(+) (*)	(1:640)	*Aggregatibacter segnis*

ND – not done; (+)-observed fungal elements; (*)-isolated cultures.

**Table 2:** Isolation of bacterial and fungal cultures from blood of mothers of children with ASD.

Mother of ASD Patient (M)	Age	Blood isolates		
		Bacterial agents (isolated cultures)	Yeast cultures (isolated cultures)	Elements of filamentous fungi observed in broths (+)
1 + 2/155 M	47 y.o.	−	−	(+)
3/161 M	39 y.o.	−	−	(+)
4/163 M	40 y.o.	−	*Candida parapsilosis*	(+)
5/165 M	46 y.o.	*Serratia marcescens*	*Candida parapsilosis*	(+)
6/179 M	35 y.o.	*Enterococcus faecalis*	*Rhodotorula mucilaginosa*	(+)
7/181 M	46 y.o.	*Pseudomonas aeruginosa*	*Candida parapsilosis*	(+)
8/183 M	40 y.o.	*Providencia rettgeri*	−	(+)
9/186 M	35 y.o.	−	−	(+)
10/189 M	31 y.o.	−	−	(+)
11/191 M	30 y.o.	*Morganella morganii*	−	(+)
12/196 M	36 y.o.	*Brevibacterium casei*	−	(+)
13/198 M	40 y.o.	*Pseudomonas aeruginosa Morganella morganii*	−	(+)
14/200 M	35 y.o	−	−	(+)
15/202 M	47 y.o.	−	−	(+)

(+) – observed elements of filamentous fungi in broths; (−) not observed.

### Recovery of bacterial cultures from blood through reversion of wall-deficient variants

**Figure 2:**
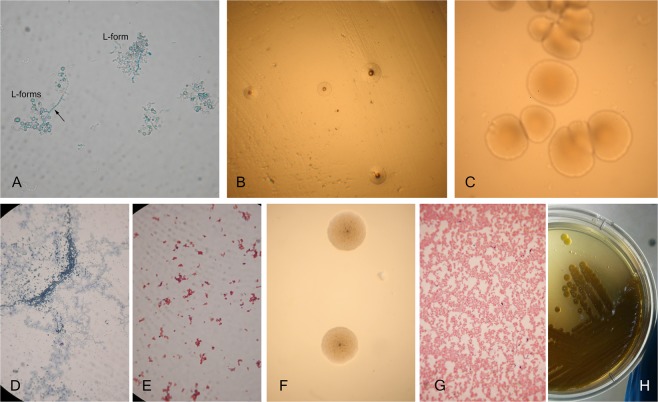
Representative L-form conversion process of *Enterococcus faecalis* and *Pseudomonas aeruginosa*, recovered from blood of autistic children: *Enterococcus faecalis* from a patient 6/178 (**A**–**C**) and *Pseudomonas aeruginosa* from a patient 7/180 (**D**–**H**). (**A**) Native preparation contrasted with methylene blue from TSB - mixed population of spherical L-forms and appearing typical chains of enterococci; (**B**) Typical “fried eggs” - shaped L-colonies on semisolid TSA; (**C**) Typical colonies of *Enterococcus faecalis* on semisolid TSA; (**D**). (**E**) Gram stained smears from L-form colonies; (**F**) L-type “fried eggs” colonies on semisolid TSA; (**G**) Gram stained smear from typical colonies of *Pseudomonas aeruginosa*; (**H**) Typical colonies of *Pseudomonas aeruginosa* on semisolid TSA. Magnification: A, D, E, G – 1000x; B, C, F- 200x.

As seen in Tables 1 and 2, L-form cultures of opportunistic bacterial species (*Enterococcus agglomerans, Rhizobium radiobacter, Enterococcus faecalis, Pseudomonas aeruginosa, Morganella morganii, Chryseobacterium indologenes*, *Brevibacterium casei* and *Aeromonas sobria*) were isolated from 8 of the children with ASD (autism spectrum disorders), as well as from 7 of their mothers (*Serratia marcescens, Enterococcus faecalis, Pseudomonas aeruginosa, Providencia rettgeri, Brevibacterium casei* and *Morganella morganii*). Bacterial cultures were not isolated from control healthy persons. The process of L-form reversion into detectable bacteria required subsequent phases of sub-cultivations and enrichment procedures in broths and semisolid media. Bacteria, recovering their cell walls, went through series of intermediate forms. As presented in Fig. 2A, a mixed population of spherical L-forms and appearing typical chains of enterococci were observed in liquid media during the phase of reversion. L-forms of *Enterococcus faecalis* grew initially as typical “fried eggs” - shaped L-colonies (Fig. 2B) but the reversion into normal bacteria terminated in formation of typical colonies of *Enterococcus faecalis* (Fig. 2B). The similar transit from “fried eggs” L-colonies into typical colonies of *Pseudomonas aeruginosa* was presented in Fig. 2(F,H). In Gram stained smears from the colonies were observed morphological forms corresponding to the phase of reversion. Gram positive granular forms (Fig. 2D) and polymorphic Gram negative forms (Fig. 2E) were characteristic for L-type colonies, while Gram negative rods corresponded to the typical colonies of *Pseudomonas aeruginosa* (Fig. 2G). Analogical phases of morphological transformation from L-forms into normal bacteria were also observed in the isolation process of other bacterial species. The isolated bacterial cultures were accurately identified by MALDI-TOF MS. The identification by MALDI-TOF MS is precise because it is based on database containing wide specter of peptide mass fingerprints (PMF) for specific genera, species and subspecies^16^.

### Recovery of fungal cultures from blood through reversion of wall-deficient variants

**Figure 3:**
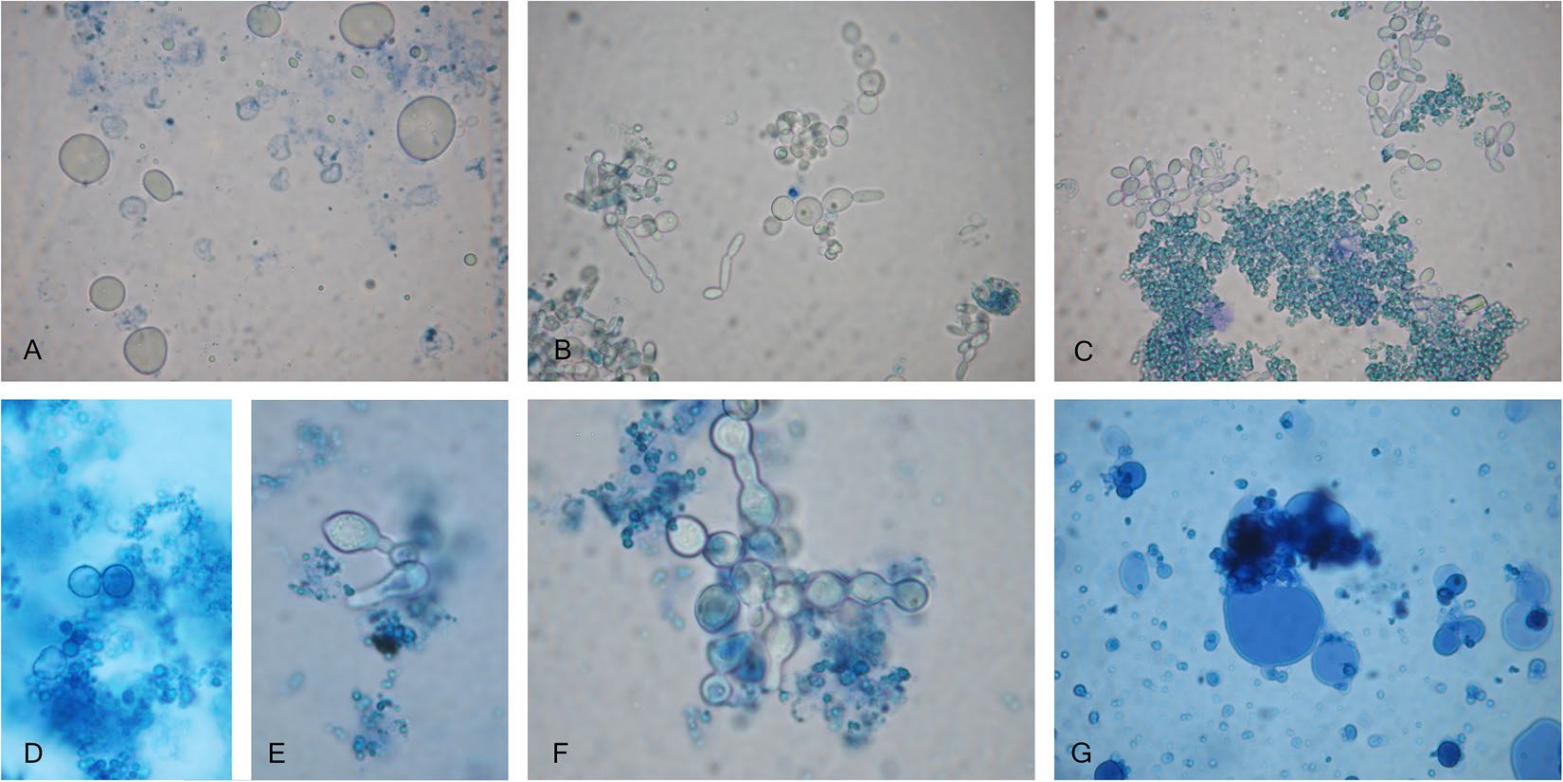
Representative conversion process of wall-deficient yeast cells. *Candida parapsilosis* (**A**–**C**), *Cryptococcus albidus* (**D**–**F**) and *Rhodotorula mucilaginosa* (**G**) recovered from blood of autistic children (patients – 1/156, 3/160, 4/162, 7/180; 11/190). Native preparations contrasted with methylene blue from SDB – A. Large protoplastic cells of *Candida parapsilosis*; (**B**,**C**). Transitional and typical cells of *Candida parapsilosis*. Magnification; 1000x.

Similar to the bacterial species, a critical factor in recovery of fungal cultures from blood was the use of a specific protocol, ensuring adaptation and development of wall-deficient forms in appropriate media (SDB and SDA) until regeneration of their wall structures, or the so called complete reversion. After reversion, their isolation and identification became possible. Cultures of *Candida parapsilosis* were isolated from 6 children; of *Cryptococcus albidus* from 2 children and of *Rhodotorula mucilaginosa* from one child (Table 1). From blood of four mothers were isolated cultures of *Candida parapsilosis* and *Rhodotorula mucilaginosa* (Table 2). The isolated yeast cultures were precisely identified by MALDI-TOF MS. Yeast cultures were not isolated from blood of control healthy. Wall-deficient yeast cells were recognized in native preparations from broths. As can be seen in Fig. 3(A–C), the isolation of *Candida parapsilosis* was preceded by morphological transformations of protoplastic cells. The size of wall-deficient forms of yeasts was larger than those of bacteria. The protoplastic yeast cells usually adopted a spherical shape (Fig. 3A). It can be seen in Fig. 3(B,C) that the first generation of cells arising from protoplasts varied in shape and size but the next generation was with typical yeast cell morphology. Complete reversion of *Candida parapsilosis* occurred on semisolid media. The same trend of morphological transformations was noted for *Cryptococcus albidus* and *Rhodotorula mucilaginosa*. Large spherical protoplastic cells were also found in both fungal species. (Fig. 3D–G).

**Figure 4:**
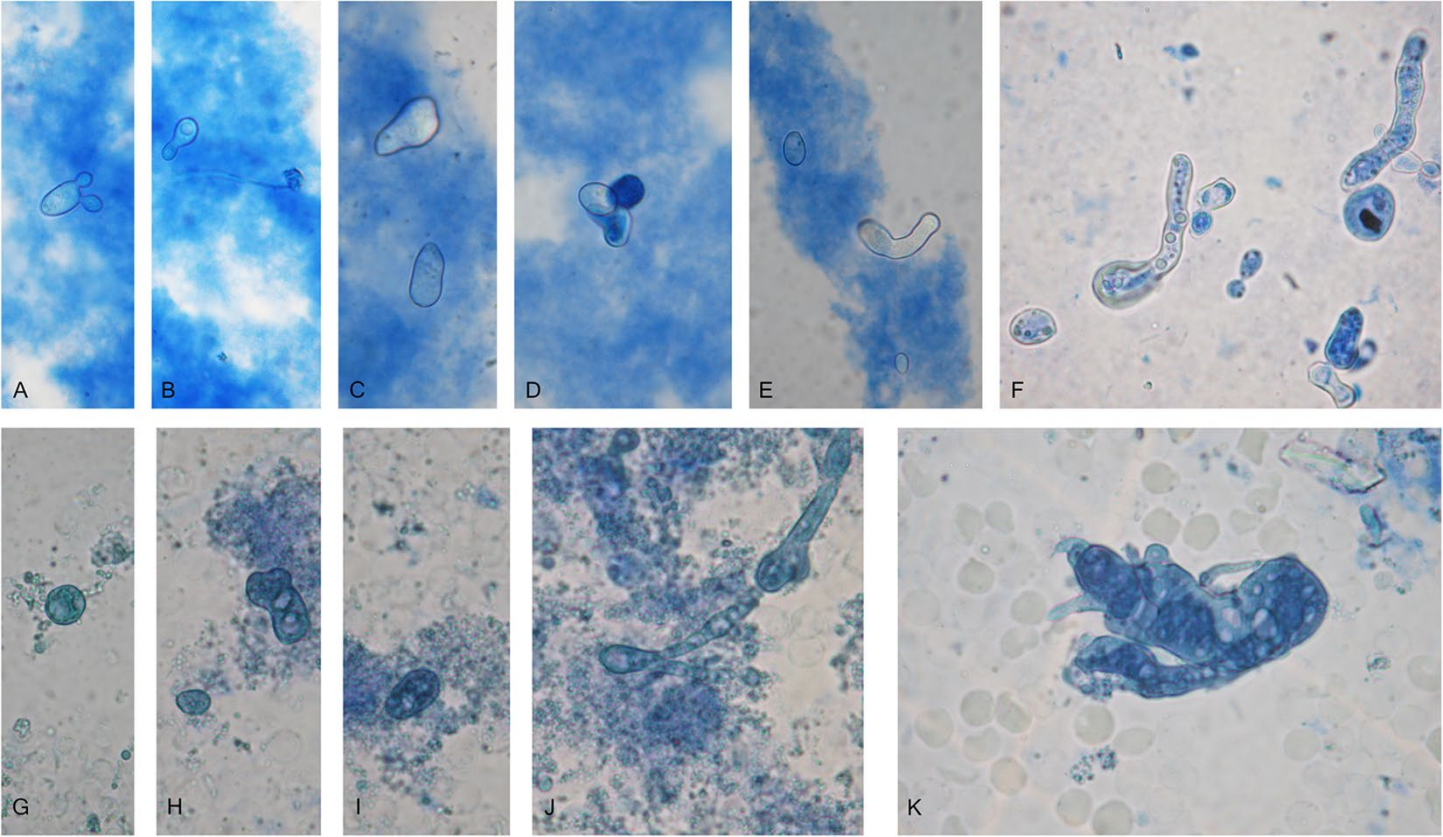
Representative pictures from conversion process of wall-deficient variant of filamentous fungi observed in SDB cultures from blood of autistic children and their mothers (patients – 1/156, 1/155 M, 7/180; 11/190, 11/191 M). Native preparations contrasted with methylene blue from SDB. Large spherical, ovoid or irregular triangle-like protoplastic cells (**A**–**E**), Process of wall restoration and conversion into a mycelial phase. (**F**–**K**) Magnification: 1000x.

**Figure 5:**
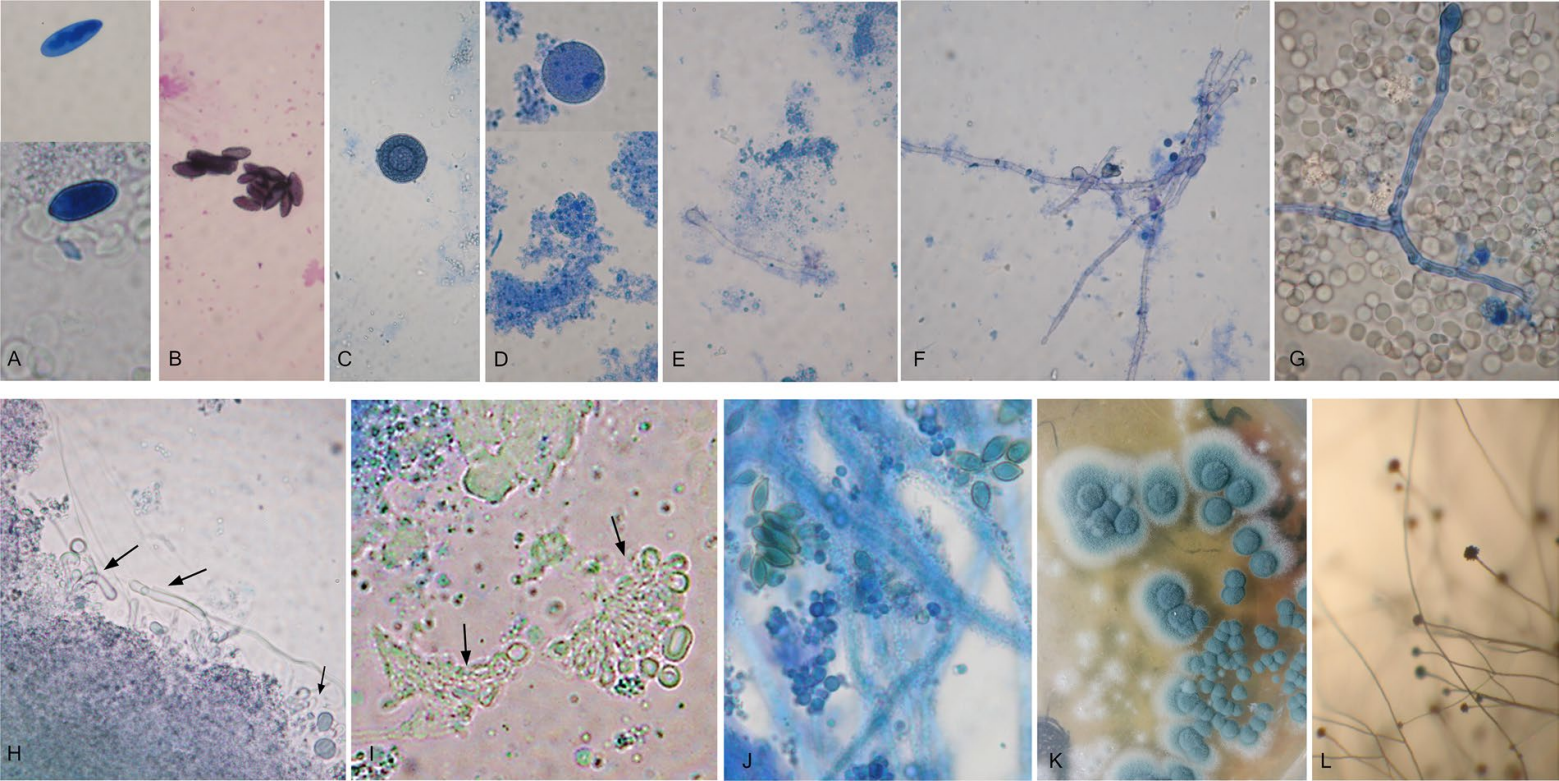
Observation of fungal elements from life cycle of filamentous fungi in SDB from blood of autistic children and their mothers. (**A**–**J**) Isolation of typical mold culture of Aspergillus fumigatus (**K**,**L**). (**A**,**B**) Single or groups of conidia. (**E**–**G**) Germination of conidia and formation of hyphae. (**C**,**D**) Fruiting bodies of cleistothecium type. (**H**,**I**,**J**) Morphogenesis of developing from protoplasts mycelial culture in SDB. Oval and elongated protoplasts were formed, further transformed into walled cells and arranged in structures resembling aspergillus heads. (**K**,**J**) After subsequent sub-cultivation on semisolid media, growth of *Aspergillus fumigatus* colonies with typical conidiophores. Magnification: (**A**–**J**) – 1000x. (**K**,**L**) – 200x.

In contrast to yeasts, the gold standard in detection of filamentous fungi remains still microscopic visualization, as well as recovery of fungal culture with typical characteristics. Seeing fungal elements in preparations from liquid media by direct microscopy provide the first signs of fungal presence in blood of autistic children and their mothers. It should be noted that fungal elements, cultivated from blood, were initially wall-deficient variants. As shown in Fig. 4(A–E), large spherical, ovoid or irregular triangle-like protoplastic cells, some of them budding, were recognized in liquid media. It can be seen during subsequent sub-cultivation that these forms started a process of cell wall restoration and conversion into a mycelial phase (Fig. 4F–K). They can form septate hyphae from several loci (Fig. 4J) or large amorphous, aseptate mycelium with vacuoles (Fig. 4K). Single or groups of conidia were observed as well (Fig. 5A,B). Conidia were seen to germinate, producing initially a tube and subsequently hyphae (Fig. 5E–G).

Other interesting findings were the closed fruiting bodies of cleistothecium type (Fig. 5C,D). These bodies, also known as cleistocarps, develop as survival structures under certain conditions. They contain asci with scattered arrangement. Ascospores are formed in an ascus by a process known as free cell formation. The mature ascocarp in Aspergillus is a round body about 100–200 μm in diameter with smooth walls. Morphogenesis of developing from protoplasts mycelial culture in liquid medium can be seen in Fig. 5(H–J). Oval and elongated protoplasts were formed, further transformed and arranged in structures resembling aspergillus heads. Subsequent sub-cultivation on semisolid media gave rise to development/growth of mold colonies, confirming the viability of the observed fungal elements in blood. Typical growth of *Aspergillus fumigatus* on semisolid medium was presented in Fig. 5(K,L). As seen in Table 1 and Table 2, in all autistic children and their mothers were found morphological elements from life cycle of filamentous fungi during microscopic observations of preparations from liquid media. In control healthy people fungal elements were not detected. Subsequent sub-cultivation on semisolid media gave rise to development/growth of mold colonies confirming the viability of the observed fungal elements in blood. Moreover, an increased titer of specific antibodies (IgG, IgM, IgA) against *Aspergillus fumigatus* was found in almost all children with ASD (Table 1).

**Figure 6:**
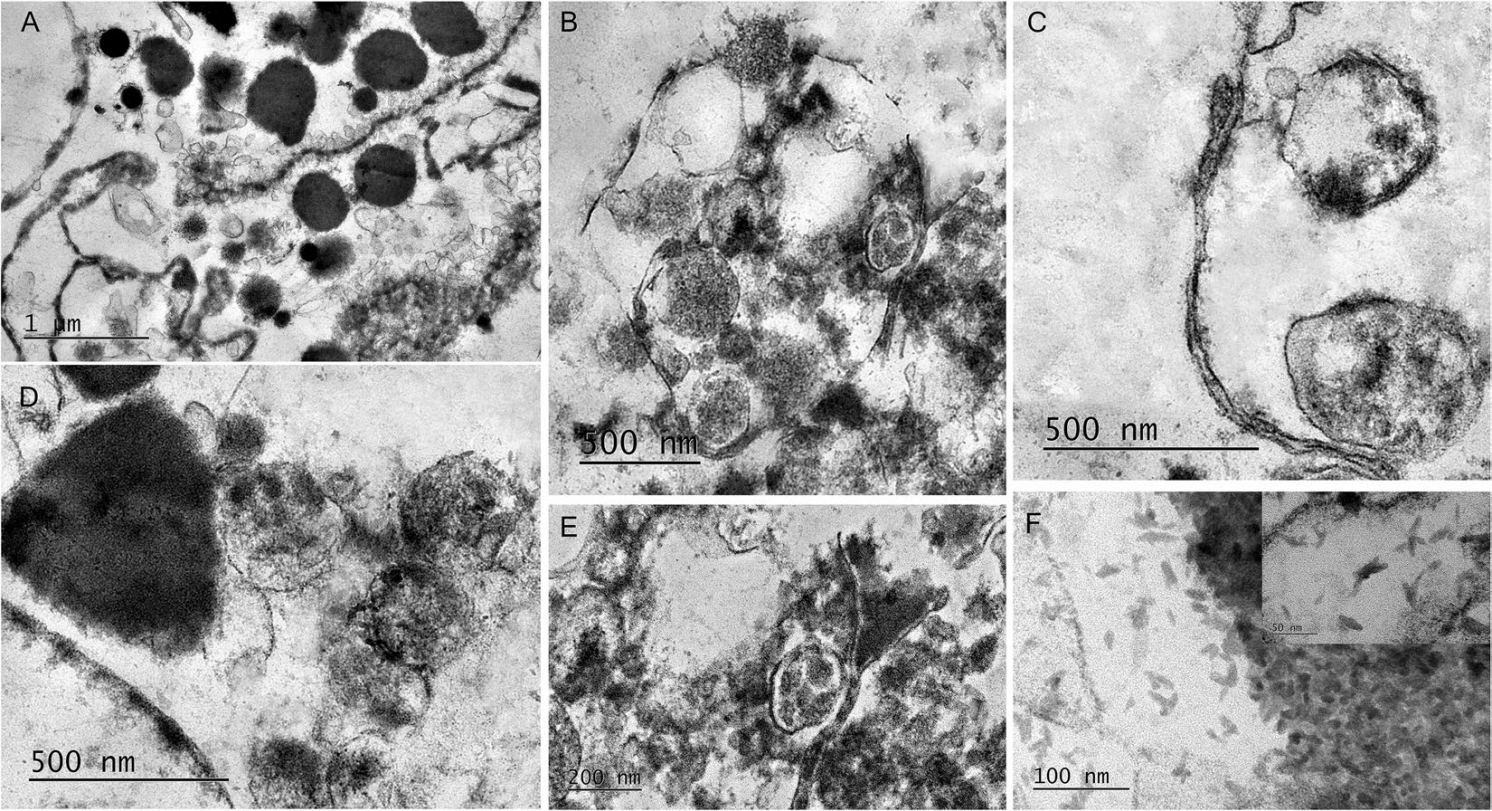
Ultrastructure morphology of developing microbial population during cultivation of blood from a child with ASD (1/156) in SDB. Transition of cell wall-deficient variants into cells with partially recovered walls. (**A**) Electron dense L-bodies of different size, as well as very small granular forms with diameter of about 100 nm, located among thickened, electron-dense and discontinuous membranous structures (regenerating wall fragments); (**C**) Ultrastructure of two cells with partly recovered walls together with a fragment of regenerated, thickened and double-contoured wall; (**B**) “Mother cell” (MC) and within it - elementary bodies and empty vesicles of varying sizes. At the top of the MC – in process of extruding a granular body. (**B**,**E**) Multinuclear and thick walled body located inside the MC, resembling fungal ascus with ascospores; (**D**) Wall-free, triangle-shaped cell, distinctive for protoplasts of fungal origin together with typical spherical L-bodies. (**F**) Abundance of nanoparticles with sizes smaller than 50 nm.

The ultrastructure morphology of developing microbial population during cultivation of blood from a child with ASD (1/156) in adapted for fungi liquid medium (SDB), was examined by transmission electron microscopy (Fig. 6). Transformation of blood L-forms (cell wall-deficient variants) into cells with partially recovered walls was a distinctive finding of main notice. In Fig. 6A, are seen electron dense L-bodies of different size, as well as very small granular forms with diameter of about 100 nm, known as “filterable forms”. The L-bodies are located among thickened, electron-dense and discontinuous membranous structures (regenerating wall fragments). Vesicular particles, structural components of plasma membrane, probably participating as building blocks in a process of wall recovery, are seen to cover the surface of a membranous structure. In Fig. 6C, ultrastructure of two cells with partly recovered walls together with a fragment of regenerated, thickened and double-contoured wall are presented. Of special interest was the observed mother cell (MC) which is often present in L-form population (Fig. 6B). MC contains elementary bodies of varying size and empty vesicles. A process of extruding a granular body is observed at the top of the MC (Fig. 6B). Particular attention should be paid to the located inside the MC multinuclear and thick walled body, resembling fungal ascus with ascospores (Fig. 6B,E). Together with typical spherical L-bodies was seen a larger cell with triangle-like shape, distinctive for protoplasts of fungal origin (Fig. 6D). Remarkably, TEM allowed to be recognized nanoparticles with size smaller than 50 nm. As seen in Fig. 6F, these particles were abundantly present in the observed cell population, even forming somewhere dense layers. TEM of L-form population from blood of а healthy person (C6/157) was done as a control. Spherical L-bodies were observed but no other morphological findings of fungal origin (ascus –like structures and nanoparticles) were detected.

### Microbial isolates from urine

From urine of 6 children were isolated bacterial cultures of Enterococcus faecalis, Pseudomonas aeruginosa, Morganella morganii, Proteus mirabilis, Acinetobacter spp., Aggregatibacter segnis, as well as yeast cultures of Rhodotorula mucilagenosa and Candida parapsilosis (Table 1).

## Discussion

The accumulated knowledge of microbial cell wall-deficient variants, or the so called L-form phenomenon, gives reason to believe that a community from L-forms could constitute a microbiota in the human blood^3–6^. The lack of cell walls and unusual biological properties make possible the survival of L-forms under bacteriostatic environment in blood^17–24^. Mattman provides own data and such of other authors, and believes that in a variety of situations, bacteria and fungi exist and grow as wall-deficient organisms, sometimes as a natural event, sometimes induced by antimicrobial agents^25^. Domingue suggests L-forms have the potential for unlimited growth and, depending on the received stimulus, they develop along different routes^8^. So, L-forms are characterized as undifferentiated and multipotent cells. Transition of L-forms into more differentiated cells has been well demonstrated by electron microscopy in our previous studies^3–6^. As it was found in the current study, a population of spherical L-forms developed from blood of healthy people, as well as from blood of autistic children and their mothers. In contrast to healthy controls, blood L-forms from autistic children and their mothers converted under appropriate conditions of cultivation into detectable bacteria and fungi. Thus, it was demonstrated that the isolated opportunistic bacteria, yeast and filamentous fungi are capable of not only existing as cell wall-deficient variants (CWD) in blood but also to revert to normal reproducing cells by synthesis a new cell wall. Recovery of bacterial and fungal cultures from blood is generally particularly difficult, especially when they are in cell wall-deficient state and it cannot be achieved by routine methods, as used in standard microbiological laboratories. However, the use of innovative methodology allowed their effective observation, cultivation, reversion, isolation and identification. It becomes clear from the results that the common, important characteristic for the children with ASD was the persistence of fungal and bacterial cell wall-deficient variants in their blood. The similar phenomenon was found in the blood of their mothers. As demonstrated in Figs 2–6, convincing evidences of CWD conversion into detectable bacteria and fungi, were obtained from light microscopic and TEM observations during the phases of their cultivation.

Based on the hypothesis for existing microbiota of cell wall-deficient variants in human blood, it can be distinguished into two types of states: (i) “eubiotic”, or balanced blood microbiota in healthy individuals and (ii) “dysbiotic”, or destabilized blood microbiota in autistic children and their mothers. When analyzing the microbiota of children and mothers, it should be noted the most obvious and unifying finding for them - the presence in blood of wall-free variants from life-cycle of filamentous fungi. As it was demonstrated in the current study, cultivation in adapted for fungi liquid media and further subcultivation on semisolid media led to development of mold colonies resembling these of Aspergillus. A positive serum antibody test, respectevely an elevated titer of specific IgG, IgM, IgA found in almost all children, was a decisive argument for proven *Aspergillus fumigatus*. No less surprising was the presence of wall-free variants of *Candida parapsilosis*, *Cryptococcus albidus* and *Rhodotorula mucilaginosa* in blood of the investigated children with ASD and some of their mothers. In support of our findings, studies of other authors have confirmed that yeast and filamentous fungi are able to exist as cell wall-deficient forms under certain conditions^26–31^.

The question arises as to whether and how presence and persistence of cell wall-deficient variants (opportunistic bacteria, yeast and filamentous fungi) in blood can contribute to autism. When, how and from what sources this phenomenon may arise in children with ASD. Various risk factors such as prenatal, perinatal and postnatal infections or exposure to environmental toxins after birth have been discussed for development of autism^32^. A study of autism cases has found a link with maternal bacterial infections in the second trimester of pregnancies^33^. When analyzing mothers’ blood microbiota in the current study, foremost stands out the fact that all of the mothers are carriers of filamentous fungal wall-free variants. From their blood were also isolated cultures of *Candida parapsilosis* and *Rhodotorula mucilaginosa*, as well as cultures of opportunistic bacteria which are often found as causes of urogenital infections. Thus, the mother’s blood microbiota can be considered as a “dysbiotic”. What is probable is that the mothers, during their adulthood, acquire and maintain long-lasting chronic bacterial infections, as well as secondary interference of fungal agents. From local focuses of chronic infections, bacteria and fungi as wall-deficient variants can enter into blood circulation, overcoming the anatomical barriers. The relationship between autistic children and their mothers should be sought during pregnancy. The pregnancy may turn out a key milestone for entrance into embryo of mother’s bacterial and fungal L-forms. Recently, we reported that persisting in human blood filterable, self-replicating L-bodies with size of 100 nm are able to cross the maternal-fetal barrier, enter fetus blood circulation and colonize newborns^3,4,6^. It seems that mother’s dysbiotic blood microbiota can be acquired by embryo as early as during ontogenetic development. This explains why children of these mothers may exhibit practically innate tolerance to wall-free variants of opportunistic bacteria and fungi.

*Candida spp*. are the most commonly diagnosed causative agent of pediatric bloodstream yeast infections, while *Aspergillus spp* are leading causes of systemic mold infections^34–36^. The diagnosis of fungal infections is always difficult to establish because in the majority of cases cultures are negative^25^. Congenital fungal infections are often overlooked and their effects on newborn health are not recognized^37^. The results from the current study give reason to think that in both autistic children and their mothers can be suspected a phenomenon of “fungal colonization” or “silent infection”, where the “carriers” experience no symptoms required for a clinical diagnosis. However, standard criteria to distinguish colonization from active infection have not been established yet. It is essential that in the case of the mothers, fungal invasion and colonization occur during their adulthood and they are usually secondary events after bacterial infections or other causes. In autistic children, fungal wall-free variants can be acquired from mothers by vertical pathway before birth and thus the newborn can be born already colonized with fungi. As was mentioned above, filterable L- forms are able to pass through the placental barrier and colonize the fetus^4,6^. In support of this assumption, were the demonstrated by TEM filterable forms in broth culture of an autistic child.

Unlike mothers, in autistic children the “fungal colonization” or “silent fungal infection” is a primary leading state during the early childhood and can strongly influence development of the immune and nervous systems. Persisting fungi remain metabolically active and can produce mycotoxins and other byproducts. Fungal metabolites are often found in the urine of autistic children and serve as markers of fungal presence^38^. It is known that in order to evade the body’s defenses *Aspergillus fumigatus* releases gliotoxin to suppress the immune system. Gliotoxin is an inhibitor of T-cell activation and of macrophage phagocytosis, as well as it induces apoptosis in monocytes and in monocyte-derived dendritic cells^39,40^. Obviously, the primary Aspergillus-induced immune suppression in children can lead to secondary polymicrobial invasion of opportunistic bacteria and other fungal species as *Candida parapsilosis* or *Cryptococcus albidus*, as it was found here. Byproducts of mold metabolism have negative effects on structural or functional integrity of developing nervous system^41^. It is known that Aspergillus secretes enzymes and proteins in large amounts and can generate nanoparticles extracellularly^42^. As it was demonstrated by TEM in the current study, an abundance of nanoparticles was found in a child with ASD, but not in a control healthy person. Fungal nanoparticles can be an effective sorbent material for toxic metals such as Al, Sb, Ba, Hg, Pb, Cd, and Tl^43^. Moreover, nanoparticles possess the property to penetrate a huge number of organs and thus to increase the toxic effects of heavy metals. Increased levels of heavy metals have been often found in autistic children^44^. It should be interesting to note that the found nanoparticles in the investigated by TEM autistic child (1/156) coincided with detected high levels of Pb, Al, Ba and Sb in his urine (data provided by parents). According to the information provided by some of the parents (2/158; 6/178, 7/180, 11/190), increased levels of heavy metals (Al, Sb, Ba, Pb, Cd, As, Tin, W, Sn) have been found in urine of their children. The relationship between production of nanoparticles (from Aspegillus fumigatus) and the detected high levels of heavy metals in autistic children is a phenomenon that deserves further and deeper investigation in order to decipher the pathogenesis and find the right pathway to treatment.

A spectrum of effects and disturbances elicited by *A. fumigatus* are primarily dependent upon the reaction of host’s immune system and may vary in wide from asymptomatic to critically ill state. However, the all spectrum of these states resides under the same diagnosis – aspergillosis, analogically to the spectrum of neurodevelopmental disorders in autistic children.

In conclusion, cell wall-deficient variants of opportunistic bacteria and fungi were recovered from blood of autistic children and their mothers. CWD converted under appropriate conditions of cultivation into detectable bacteria and fungi. The unifying finding for autistic children and their mothers was the presence in blood of wall-free variants from life-cycle of *Aspergillus fumigatus*, a phenomenon of fungal “colonization” or “silent infection”. It can be assumed that autistic children may be born with fungal colonization acquired from mothers by transplacental pathway. “Silent aspergillosis” may strongly influence development of immune and nervous systems in the early childhood and be a leading cause for neurodevelopmental disorders.

A promising area for future research is development of criteria for personalized evaluation of blood microbiota and early screening of microbial colonization in newborns and their mothers, as well as selective approach to treatment and care of these newborns in order to prevent development of autism.

## Materials and Methods

### Study scheme

As listed in Tables 1 and 2, fifteen children (ages 3–12 years) with diagnosis for autism spectrum disorder, as well as their mothers (ages 30–47 years) were studied. The diagnosis of children has been done according to the standard international criteria. As controls were studied 6 healthy persons-C1/31 (male 18 y.o.), C2/51 (male 20 y.o.), C3/52 (male 22 y.o.), C5/81 (male 16 y.o.), C5/134 (female, 12 y.o.), C6/157 female 17 y.o.) Blood samples were aseptically collected from all investigated persons using K2E-EDTA Vacutainer tubes (BD Vacutainer, Plymouth, UK). Informed consent for the use of the blood samples for research purposes was obtained from all participants and/or their legal guardians. All blood samples were handled and anonymized, according to the national ethical and legal guideline and the study protocol was approved by the Ethics Committee of Scientific Studies Involving Human Experimentation at the Medical University of Sofia.

### Isolation of blood L-forms and conversion into bacterial and fungal cultures

For isolation of microbial L-type cultures from blood samples was used two protocols designated as “classical” and “filtration” which is described in our previous studies^3,4^. In brief, blood lysis was done with sterile distilled water at strictly fixed v/v ratio and after 30 min exposure to room temperature. According to the “classical” protocol (CL) the aliquots from lysed blood samples were inoculated in tubes with Tryptic Soy Broth (TSB, Becton Dickinson) and incubated at 37 °C for 72 hours. According to the “filtration” protocol (F), after inoculation TSB was filtered through a bacterial filter with 0.2 µm size of pores and was incubated also at 37 °C for 72 hours. Then, strictly fixed aliquots from primary broths (CL and F) were sub-cultured again in three variants of broth media (TSB, TSB with Gentamycin of 100 µg/ml and Sabouraud Dextrose broth- SDB with Chloramphenicol of 50 µg/ml) and parallel plated on three variant of semisolid media -TSA, TSA with Gentamycin of 100 µg/ml and Sabouraud Dextrose Agar-SDA with Chloramphenicol of 50 µg/ml. The semisolid media were solidified with 0.8% (w/v) Agar (Fluca). TSB and TSA were incubated at 37 °C, while SDB and SDA at 25 °C. Passages in broth and semisolid media were performed using technique described in previous study^3^. In control experiments for the sterile performance of the technical procedures, broths and semisolid media were inoculated with sterile saline and subsequent transfers were done by the same technique. All cultures were periodically observed for appearance of growth and morphological transformations within 2 months. Direct light microscopic observations of native preparations from cultures were combined with Gram and Giemsa stained smears. The isolated pure bacterial and yeast cultures were identified by matrix assisted laser desorption ionization-time of flight mass spectrometry technology (MALDI-TOF MS) using intact cells. This tool for microbial (bacteria and fungi) identification is based on automation in proteomics technology. Identification of microbes by MALDI-TOF MS is done by comparing the characteristic spectrum called peptide mass fingerprints (PMF) of unknown organisms with the PMFs contained in the database. This technology is already applied worldwide for microbial identification, by using commercial libraries of organisms PMFs^16^. MALDI-TOF MS equipment (Vitek MS - BIOMERIEUX) was used in the current study and all procedures were done according to the protocol of the manufacturer by trained laboratory personnel in a national public health laboratory. In parallel to blood study, urine from children with autism was microbiologically investigated following standard techniques.

### Serological test

*Aspergillus fumigatus* –specific IgA, IgG, IgM were determined by standard serum haemagglutination test.

### Transmission electron microscopy (TEM)

Observations of broth L-form culture from blood of child with ASD (1/156) was performed by electron microscopy. A depot from broth culture was harvested by centrifugation at 3000 rpm for 20 min. After that, the depot was fixed with 4% (v/v) glutaraldehyde in 0.1 M cacodylate buffer with 4.5% w/v sucrose, pH 7.2 and post-fixed in 1% (w/v) osmium tetroxide in the same buffer at room temperature for 2 h and dehydrated in serial ascending ethanol concentrations. After dehydration in ethanol and propylene- oxide series, cell pellets were embedded in epoxy resin Epon-Araldite (Serva, Heidelberg, Germany). Resin blocks polymerized at 56 °C for 48 h. Ultrathin cell sections were made with crystal glass knives on a Reichert-Jung Ultracut Microtome and were stained with 5% (w/v) uranyl acetate in 70% (v/v) methanol and 0.4% (w/v) lead citrate. Observations were made with electron microscope JEOL JEM -1011 SAP10 (Japan) at 40–100 kV.
